# Adipose-Derived Stem Cells to Treat Ischemic Diseases: The Case of Peripheral Artery Disease

**DOI:** 10.3390/ijms242316752

**Published:** 2023-11-25

**Authors:** Gemma Arderiu, Anna Civit-Urgell, Lina Badimon

**Affiliations:** 1 Institut de Recerca de l’Hospital de la Santa Creu i Sant Pau, IIB-Sant Pau Barcelona, 08041 Barcelona, Spain; acivit@santpau.cat (A.C.-U.); lbadimon@santpau.cat (L.B.); 2 Ciber CV, Instituto Carlos III, 28029 Madrid, Spain; 3 Facultat de Medicina i Ciències de la Salut—Campus Clínic, Universitat de Barcelona, 08007 Barcelona, Spain

**Keywords:** peripheral artery disease, critical limb ischemia, adipose-derived stem cells, cell therapy, angiogenesis, ASC differentiation

## Abstract

Critical limb ischemia incidence and prevalence have increased over the years. However, there are no successful treatments to improve quality of life and to reduce the risk of cardiovascular and limb events in these patients. Advanced regenerative therapies have focused their interest on the generation of new blood vessels to repair tissue damage through the use of stem cells. One of the most promising sources of stem cells with high potential in cell-based therapy is adipose-derived stem cells (ASCs). ASCs are adult mesenchymal stem cells that are relatively abundant and ubiquitous and are characterized by a multilineage capacity and low immunogenicity. The proangiogenic benefits of ASCs may be ascribed to: (a) paracrine secretion of proangiogenic molecules that may stimulate angiogenesis; (b) secretion of microvesicles/exosomes that are also considered as a novel therapeutic prospect for treating ischemic diseases; and (c) their differentiation capability toward endothelial cells (ECs). Although we know the proangiogenic effects of ASCs, the therapeutic efficacy of ASCs after transplantation in peripheral artery diseases patients is still relatively low. In this review, we evidence the potential therapeutic use of ASCs in ischemic regenerative medicine. We also highlight the main challenges in the differentiation of these cells into functional ECs. However, significant efforts are still needed to ascertain relevant transcription factors, intracellular signaling and interlinking pathways in endothelial differentiation.

## 1. Introduction

Since the last century, cardiovascular diseases (CVDs) have been the leading cause of death and are also a major contributor to premature death, disability, health care expenditures, and lost productivity globally [1,2,3]. CVDs are a set of heterogeneous chronic heart and circulatory system disorders that are asymptomatic; only advanced disease causes multifactorial symptoms, and gradually evolves throughout life [4]. This group of disorders includes coronary heart disease, cerebrovascular disease, peripheral artery disease (PAD) and other vascular conditions [2].

PAD is a vascular disease caused mainly by atherosclerosis. It is defined as the narrowing and obstruction of the blood flow of major systemic arteries other than those of cerebral and coronary circulations [5]. Symptomatic clinical manifestations of PAD include intermittent claudication (IC) and critical limb ischemia (CLI), which is the most severe manifestation. Patients with CLI have a major risk of tissue loss, amputation, cardiovascular events and mortality [5,6,7,8]. Treatment for patients with CLI is usually directed toward limiting the consequences of systemic atherosclerosis, such as is the case with myocardial infarction or stroke. Therefore, therapies such as statins, angiotensin converting-enzyme inhibitors or angiotensin-receptor blockers, and antiplatelet agents are frequently used [9,10]. All patients with ischemic ulcers have to receive wound care and other medical approaches to promote healing and reduce pain. Of note, no medical therapies are effective in improving perfusion to the lower extremity in patients with PAD. Surgical and catheter-based revascularization are the preferred approaches for CLI and should be considered the first treatment option. In correctly selected patients, either modality can result in preservation of life and limb of ≥75% at 1 year. However, although numbers vary greatly from center to center, many patients with CLI are poor candidates for revascularization or have no option for revascularization at all. Even after a successful revascularization, the graft can fail, or stenosis can reoccur after catheter-based treatment. Clearly, a need exists to develop new treatment strategies for patients with CLI who are not candidates for revascularization [11,12].

The concept of therapeutic angiogenesis has emerged as an investigational approach to identify angiogenic agents to promote the development of collateral vascular networks in ischemic tissues and subsequently increase perfusion [13]. Angiogenesis is the development of new blood vessels from the preexisting ones. These remodeling and growth of new vessels can be achieved by gene therapy, protein therapy and cell therapy. Cell-based therapy is focused on the use of stem cells due to their unlimited therapeutic potential [14]. Stem cells are resident cells in the bone marrow, where three different populations co-exist: endothelial progenitor cells, bone-marrow mesenchymal stem cells and hematopoietic stem cells. Stem cells can also be found in tissues (also referred as somatic stem cells or adult stem cells) as in white adipose tissue, skeletal muscle and myocardium. 

Stem cells exhibit self-renewal capacity and can differentiate into multiple cell phenotypes that offer therapeutic solutions for many diseases [15]. As such, a novel therapeutic option is the delivery of autologous or allogenic adult stem cells into ischemic tissue to prevent tissue damage in the affected area [16]. One of the most promising sources of stem cell populations with high potential in clinical medicine is adipose-derived stem cells (ASCs). ASCs are adult mesenchymal stem cells isolated from white adipose tissue that exhibit different interesting properties. Numerous reports underline the capacity of ASCs to differentiate into other cell types, including endothelial cells (ECs) [17,18]. This multilineage capacity together with their abundance and low immunogenicity lead to the supposition that these cells have a prominent role in cell-based therapy [15]. ASCs have been deeply studied in the field of neovascularization. ASCs secrete multiple angiogenic and antiapoptotic biological molecules, such as growth factors [19], cytokines [15], microvesicles (MVs) and proangiogenic molecules, that participate in the formation of neovascular-like structures and interact with microvascular ECs [20]. The ability of ASCs to differentiate into ECs has been described as a potential mechanism for the ASC-mediated pro-angiogenic benefits. However, key transcription factors, intracellular signaling, and interlinking pathways that promote ASC differentiation toward specific lineages or cell fates are still under investigation.

Hence, the aim of this review is to rigorously evaluate the relevant scientific literature for conclusive evidence that ASCs can differentiate into functional ECs and contribute to vascular repair and clinical improvement in CLI.

## 2. Peripheral Artery Disease

The aorta, the main artery of our body, is bifurcated into two arteries, which at the point of the leg are branched into several arteries, but with one main artery supplying blood flow to the distal leg. The formation of an atherosclerotic plaque disrupts blood flow and results in vessel restriction or occlusion. There is an interruption/cessation of the vessel metabolic demands that leads to limb ischemia. The formation of reactive oxygen species results in cell dysfunction, or even cell death, of blood vessel-forming cells and perpetuates tissue ischemia [7,21]. PAD is the pathological condition that results in the obstruction of vessels and obliteration of arterial blood flow, limiting blood supply to organs other than the heart [7,21,22]. PAD is a common disease; it is the third leading cause of atherosclerotic cardiovascular morbidity and often remains unrecognized and underdiagnosed. Traditional CVDs risk factors are associated with the risk of developing PAD: age, cigarette smoking, diabetes mellitus, hypertension, hypercholesterolemia, and sedentary state. All these major atherosclerotic risk factors are associated with a 2- to 4-fold increased risk of PAD [7,22]. PAD is classified as: (a) asymptomatic, where the lack of noticeable symptoms puts patients at risk of morbidity and mortality; or (b) symptomatic, where the degree of manifestation ranges from intermittent claudication to CLI. Intermittent claudication is a primarily quality-of-life disease characterized by the occurrence of leg pain, arching, cramping, or fatigue induced by exercise that can be relieved by resting. Meanwhile, as the disease worsens, the aggravation of the lesion leads to CLI, and it is characterized by chronic ischemic rest pain present for at least 2 weeks with or without ulcers or gangrene [7,21].

## 3. Critical Limb Ischemia

**Figure 1:**
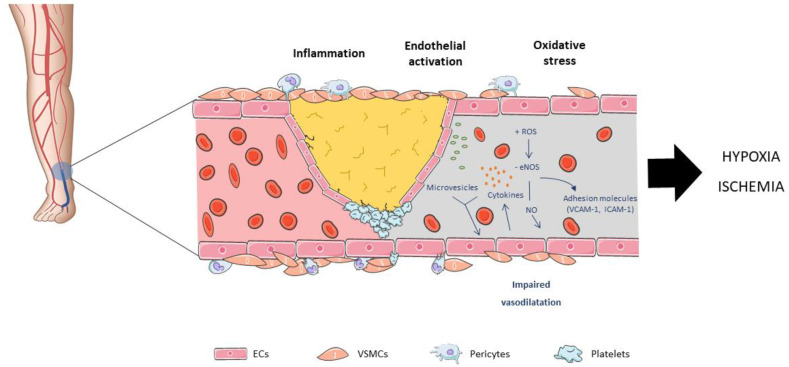
Peripheral arterial disease (PAD) is mainly caused by atherosclerosis. The earliest event is endothelium activation by adherence of mononuclear cells to endothelial cells (ECs), followed by an inflammatory cascade, the formation of fatty streaks and leading to atheroma plaque formation. Endothelial dysfunction entails a change in the synthesis and secretion of different substances and expression of diverse endothelial genes. Among them, monocyte chemoattractant protein-1, platelet-derived growth factors (PDGFs), platelet adhesion molecule-1, or endothelial nitric oxide synthase (eNOS). In the presence of hyperlipidemia, hypertension, smoking or diabetes, there is an increased oxidative stress, which promotes the synthesis of pro-atherogenic molecules such as cytokines and chemokines, interleukins, adhesion molecules, leading to an inhibition of eNOS activity, and consequently a reduction in nitric oxide bioavailability.

CLI represents the most advanced form and end stage of PAD, which results in the occlusion of the arteries, leading to hypoxia and ischemia of the skeletal muscle. It is a highly morbid disease with a diminished health-related quality of life associated with a high risk of tissue loss, limb amputation and a precursor of cardiovascular events (Figure 1).

The goals of medical management for CLI are not only to improve quality of life but also to reduce the risk of cardiovascular and major limb events. It includes aggressive risk factor modification, including lifestyle modification, smoking cessation, antithrombotic and antihypertensive therapy and lipid-lowering therapy [7,22,23]. However, bypass vascular surgery and endovascular interventions are necessary to restore vascular function and structure and revascularization is the optimal treatment for CLI to increase limb perfusion. Even after successful revascularization procedures, residual microvascular disease may well limit the effectiveness of the interventions [24]. Moreover, a significant proportion of patients (up to 1 of 3) cannot benefit from this intervention because size and severity of the lesion, which finally leads to limb amputation or palliation [22,23]. There is a necessity to formulate novel treatment approaches for individuals diagnosed with CLI who are ineligible for revascularization.

In the last decades, angiogenesis has appeared as a hope for these patients. This complex process leads to new blood vessel formation and acts as a compensatory mechanism in response to ischemic diseases. It increases oxygen and nutrients supplies to tissue in order to protect their function.

## 4. Therapeutic Angiogenesis

Efficient and simultaneous transport of gases, liquids, nutrients, signaling molecules and circulating cells to organs is necessary to meet the metabolic demands of active cells [24,25]. Homeostasis is maintained by complex and highly branched tubular networks of vessels integrating the vascular system. The arterial wall is a three-layered structure composed of the tunica intima, media and adventitia and finally surrounded by perivascular adipose tissue. The tunica intima, which is in intimate contact with perfusing blood is composed by a single layer of endothelial cells (ECs). Vascular smooth muscle cells (VSMCs), collagens and elastic fibers are the main components of the tunica media. Tunica adventitia is composed of connective tissue, progenitor cells, fibroblasts, adipocytes, nerves and *vasa vasorum* [26,27]. All these cell types and protein matrices are essential for maintaining stable vascular structure and function. Under pathological conditions, ischemic tissue has a low oxygen supply, free radical production, and cellular response, which induce EC dysfunction and inflammatory response. Regeneration of the vasculature requires transcriptional signaling that enhances vascular repair and remodeling, by the activation of transcription factors, such as hypoxia-inducible factor-1 [22,24]. This transcription factor stimulates the release of angiogenic factors, cytokines and other signals to induce existing capillary EC proliferation, migration, and invasion of the host stroma toward the source of angiogenic stimuli to restore the microvasculature [28]. Vascular growth can occur in three different forms that together contribute to a high level of organization and maturation of the vascular network and, also, contribute to tissue repair and remodeling in ischemic vascular diseases [25,28,29]. Angiogenesis, arteriogenesis and vasculogenesis contribute to neovascularization, as they are part of the same process and complement each other. These cellular processes are driven by partially overlapping cellular and molecular pathways [28]. Angiogenesis is defined as the sprouting of new capillaries from existing vessels, which involves temporally and spatially regulated changes in gene expression [25]. In adults, most vasculature is quiescent, and angiogenesis is required for tissue repair or remodeling. The arteriogenesis process consists on the stabilization of primitive vessels into the capillary network through the generation of an extracellular matrix by mural cells and the activation of ECs downstream signaling pathways and cytokines that mediate vessel remodeling. Adult vasculogenesis is another process of neovascularization where endothelial progenitor cells are mobilized from the bone marrow to the endothelium, differentiate into ECs, and contribute to microvasculature restoration via paracrine functions [21,28]. The interplay of these three neovascularization processes is important for restoring limb function after ischemia. However, these processes are disrupted in PAD fundamentally due to microvascular EC dysfunction [25].

The concept of ‘therapeutic angiogenesis’ emerged approximately two decades ago as an investigational approach for patients with ischemic lesions who did not undergo revascularization. This technique is based on the remodeling and growth of new vessels in the ischemic region to alleviate hypoxic damage to organs and tissues [30]. Regenerative medicine therapies include gene therapy, protein therapy and cell-based therapy. Protein therapy is based on the administration of angiogenic growth factors, such as vascular endothelial growth factor (VEGF) or fibroblast growth factor (FGF), at a specific site of interest to promote angiogenesis and collateral artery formation. However, the short half-life of these proteins, the high dosages needed, the repeated injections, and the protection from proteolytic degradation have led to investigations on gene or cell-based therapy [21,23,30]. Gene therapy consists of the introduction of pro-angiogenic genes into ischemic sites using nonviral or viral vectors. These vectors are integrated into the host chromosome, resulting in expression of angiogenic genes even after cell division. However, an overproduction of a certain angiogenic factor could inhibit blood vessel formation. Thus, it is required a precise balance between different signals [30]. Initially, the first agent transfected was VEGF isoform 165 and 121. Few years later, other pro-angiogenic factors such as FGF, hepatocyte growth factor, platelet-derived growth factor, prokineticin 2, angiogenic factor with G-patch and Forkhead-associated domain 1, human telomerase reverse transcriptase (hTERT), and some microRNAs, also showed promising results in promoting angiogenesis in animal models [13,21,31]. The objective is to maintain the angiogenic activity of the gene transcribed molecules at a specific site of interest [32]. Cell-based therapy refers to the transfer of autologous or allogenic cells, which can be genetically engineered or manipulated and can be administered topically or as injectables, infusions, bioscaffolds or scaffold-free systems. The selection of potential candidate cells is based on their capability to self-renew and differentiate into blood-vessel-associated cells or organ-specific cell types, or secretion of pro-angiogenic growth factors. Several stem cells had been identified, isolated and applied in clinical trials. However, some challenges have to be overcome: acquiring enough cells, in vivo viability and integration with the host tissue [33]. The cells that have been used in clinical trials of cell-based therapies in PAD include bone marrow mononuclear cells bone marrow mesenchymal stem cells, granulocyte colony stimulating factor-mobilized peripheral blood mononuclear cells, endothelial progenitor cells, and granulocyte colony stimulating factor monotherapy [13]. Moreover, cell-based therapy is used in multiple therapeutic areas such as regenerative medicine, immunotherapy, and cancer therapy, and it combines stem cell- and non-stem-cell-based unicellular or multicellular therapies [34]. To date, cell-based therapy in the field of regenerative medicine involve either the administration of differentiated stem cells into vascular cells or the induction of angiogenic growth factor expression through paracrine signaling exerted by the respective cell [21,23].

## 5. Stem Cells: Adipose Tissue-Derived Stem Cells 

Stem cells are an undifferentiated cell population with the ability to extensively proliferate, replicate themselves (clonate) and differentiate into different cell lineages. Stem cells are found in different tissues with different levels of differentiation (pluripotent, multipotent, and tissue-resident stem cells). These different potencies make them candidates for cell-based therapy (Table 1) [35]. 

After vascular injury, there is a process which entails ECs proliferation and migration. However, it has been reported that in response to vascular injury or dysfunction, stem progenitor cells are homed to the lesion area where they can differentiate into vascular ECs, VSMCs and inflammatory cells contributing to revascularization. Moreover, it has also been reported that there are vascular stem progenitor cells residing within the structure of the vessel wall that can differentiate into several types of vascular cells and promote angiogenesis [26,36]. These stem progenitor cells include endothelial progenitor cells, smooth muscle progenitor cells, mesenchymal stem cells and pericytes. Mesenchymal stem cells are multipotent non-hematopoietic stromal cells, that can be isolated from various adult tissues, that are considered to provide structural support to the organs [37]. These cells are considered one of the most important cell types for cell-based therapy, not only for their multilineage differentiation capacity, but also for their active secretome, that can promote autocrine and paracrine signaling contributing to angiogenesis, and to cross-talk with resident stem cells [34]. Within mesenchymal stem cells, ASCs are a multipotent mesenchymal stem cell population, relatively abundant that can be easily isolated in large quantities from adipose tissue [38,39,40,41].

**Table 1:** Studies using stem cells to treat different diseases.

Cell Source	Disease	Type of Study	Reference
Human embryonic stem cells	Diabetes mellitus	In Vivo	Kroon et al. [42]
Human adipose tissue-derived mesenchymal stem cells	Type 1 diabetes mellitus	Clinical trial	Trivedi et al. [43]
Mouse adipose tissue-derived stem cells	Type 2 diabetes mellitus	In Vivo	Wang et al. [44]
Hematopoietic stem cell transplantation	Chronic myeloid leukemia	Clinical trial	Hackanson and Waller et al. [45]
Bone marrow-derived mesenchymal stem cells	Liver failure	In Vivo	Kuo et al. [46]
Human embryonic stem cells	Pulmonary fibrosis	In Vivo	Banerjee et al. [47]
Autologous hematopoietic stem cells	Refractory Crohn’s disease	Clinical trial	Cassinotti et al. [48] Oyama et al. [49]
Autologous non-myeloablative hemopoietic stem cells	Multiple sclerosis	Clinical trial	Burt et al. [50]
Mouse embryonic stem cells	Parkinson disease	In Vivo	Björklund et al. [51]
Rat autologous adipose tissue-derived stem cells	Wound healing	In Vivo	Zhou et al. [52]
Allogenic adipose tissue-derived mesenchymal stem cells	Acute ischemic stroke	Clinical trial	De Celis-Ruiz et al. [53]
Human adipose-derived mesenchymal stem cells exosomes	Atherosclerosis	In Vitro and in vivo	Yu et al. [54] Xing et al. [55]

**Figure 2:**
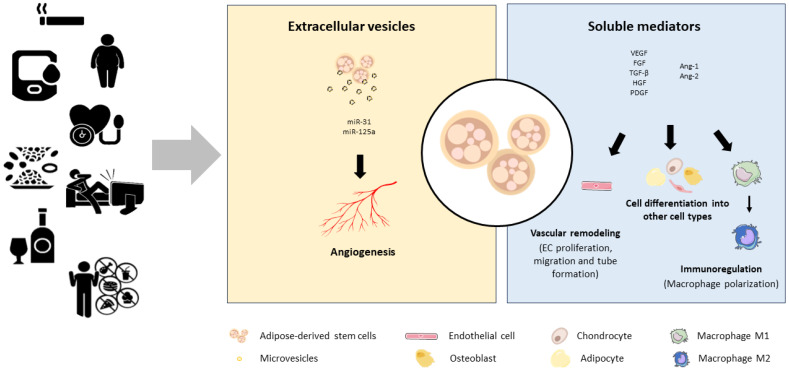
ASC autocrine and paracrine signaling. ASCs secrete many significant autocrine and paracrine proteins, such as soluble growth factors (bFGF, VEGF), microvesicles enriched with miRNAs (miRNA-31, miRNA-126, miRNA-125a) and immunomodulatory molecules that influence immediate blood capillary environment and induce ASC differentiation into multiple cell lineages. These pro-angiogeneic benefits are modified by cardiovascular risk factors such as hypertension, obesity, diabetes, dyslipidemia, smoking, physical inactivity, alcohol misuse or unhealthy diet.

The International Federation for Adipose Therapeutics and Sciences and the International Society for Cell and Gene Therapy established a minimum criterion for the characterization of ASCs: plastic adhesion, expression of different surface antigens and the in vitro potency to differentiate into pre-adipocyte, chondrocyte, and osteoblasts [56]. There is a common consensus that these cells do not express unique surface markers but do express surface antigens of mesenchymal stem cells. Immunophenotypically, ASCs can be identified by the presence (CD90, CD44, CD29, CD105, CD13, CD34, CD73, CD166, CD10, CD49e and CD59) and absence (CD31, CD45, CD14, CD11b, CD19, CD56 and CD146) of several surface markers. However, the expression of some cell surface markers remains controversial. For instance, some data sustains that ASCs can be distinguished from bone marrow stem cells by the lack of CD106 surface antigen expression [57,58]; and other surface marker, CD34, is present in freshly isolated ASCs but its expression disappears or remains at low levels after several passages [58,59]. But even though ASCs change progressively their phenotype with passages in vitro, their multilineage differentiation and proliferation capacity is retained [60]. Moreover, these cells do no express type II human leukocyte antigen and thus do not induce immunological rejection after allogenic transplantation and are exempt from ethical implications [26,37,61] as it is the case with embryonic stem cells. For this reason, these adult mesenchymal stem cells have been investigated and used in a wide variety of fields due to their commitment to differentiate into many cell types. There is reported information on their use in cardiovascular diseases, metabolic diseases, skeletal tissue regeneration or wound healing and skin aging, among others. Currently, there are more than 100 ASC clinical trials registered in the Clinical Trials database [62]. However, their secretome, microvesicles and differentiation capacity into the endothelial lineage has attracted the interest of researchers (Figure 2).

## 6. The Importance of ASCs Secretome and Its Effects on Cellular Mechanisms

ASCs secrete multiple bioactive molecules involved in cellular differentiation, migration, proliferation and autocrine and paracrine signaling [20]. The secretome of ASCs is composed by pro- and anti-inflammatory cytokines and chemokines [15], growth factors [19], cytosolic components, and extracellular vesicles, among others [63,64].

A notable mechanism associated with the development of collateral vascular networks and increased perfusion after ASCs transplantation is the secretion of multiple autocrine and paracrine angiogenic growth factors [33,65,66]. Growth factors, such as vascular endothelial growth factor (VEGF), basic-fibroblast growth factor (bFGF) or transforming growth factor (TGF)-β, bind to their receptors in the cell membrane of ECs and lead to cellular activities such as endothelial growth, migration, and tube formation [63,67]. 

Apart from soluble growth factors, further investigations by Kang et al. recognized a new mechanism of cell–cell communication. These investigations evidenced that ASCs release MVs that can directly promote angiogenesis in vitro and in vivo. MVs are vesicles that are enclosed by a phospholipid bilayer membrane, contain natural signaling molecules and act as an intercellular communicators [27]. In vivo experiments have revealed that ASC-derived MVs (ASCs-MVs) promote tube formation and significantly up-regulate the expression of growth factors and receptors on ECs [68]. These MVs are enriched with miRNAs, in particular miRNA-31, promoting migration and tube formation in recipient ECs. These pro-angiogenic effects were also observed in mouse Matrigel plug assays, which revealed that ASC-MVs induce functional vasculature formation [64]. Other investigations revealed that ASC-MVs are enriched with miRNA-125a, which promotes the formation of endothelial tip cells by repressing the angiogenic inhibitor delta-like 4 [69].

Angiogenic capacity of ASCs may be modified by different factors. ASCs may be obtained from different source of adipose tissue, subcutaneous or visceral adipose tissue. It has been reported that the metabolic differences between both depots affect ASCs properties such as proliferation, differentiation and apoptosis, as well as gene expression patterns. ASCs from the subcutaneous adipose tissue have a greater capacity to proliferate and differentiate, whereas ASCs from visceral adipose tissue express related to lipid metabolism [41,70,71]. In addition, cardiovascular risk factors also negatively modified their properties. Ferrer et al. demonstrated that ASCs pluripotency, self-renewal capacity, differentiation and angiogenic properties were modified by type 2 diabetes mellitus [72]. Obesity also impairs ASCs properties in both depots, specifically it affects genes related to stemness, lineage commitment and inflammation. ASCs from obese patients compared to ASCs derived from nonobese patients show lower proliferation, differentiation and proangiogenic capacities, overall negatively modifying ASCs regenerative capacity [41,73,74]. Diabetes mellitus is one of the main risk factors of PAD, increasing the risk of lower limb amputation. Autologous ASCs from these patients have their proangiogenic properties impaired, so allogenic ASCs are required to induce vascular remodeling.

As ASCs are an ideal cell source for angiogenic therapy and autologous cells have limitations, it is needed to increase their angiogenic potency in the presence of cardiovascular risk factors.

## 7. ASCs Differentiation into an Endothelial-like Phenotype to Increase Their Angiogenic Potential

To direct ASCs differentiation potential into endothelial lineage-specific pattern, in vitro studies are centered on manipulating the components of endothelial growth medium (EGM). EGM is usually supplemented with two angiogenic growth factors: VEGF and bFGF, both implicated in the differentiation pathway into EC-like cells. Several studies indicated that these factors are two plausible factors to induce endothelial characteristics of ASCs. VEGF is a 40 kDa extremely potent heterodimeric glycoprotein with pro-angiogenic functions. In humans, VEGF family involves several members, amongst which it must be outlined the role of VEGF-A [75,76]. This growth factor actively participates in the regulation of the angiogenesis process. It contributes to the revascularization process by the mobilization and recruitment of endothelial and hematopoietic stem cells. These stem cells express the tyrosine kinase cell receptors VEGFRs, receptors expressed predominantly on vascular ECs. The angiogenic factor VEGF binds to VEGFRs increasing vascular permeability and the migration, proliferation, and differentiation of ECs thus contributing to angiogenesis [75,76,77]. After 10 days of stimulation, ASCs change their morphology towards a EC-like morphology [78]. In addition, to confirm the induction of functional ECs in vitro, the studies observed the formation of capillary-like structures in Matrigel-coated coverslips after treatment with VEGF [18,78]. Commitment toward an endothelial lineage was further determined by significant increases in EC-specific markers such as CD31, von Willebrand Factor, CD144, and eNOS [18,77,78]. A synergistic effect of VEGF and FGF has also been proposed. ASCs cultured in medium containing FGF and VEGF show increased EC markers PECAM-1, CD34, VE-cadherin, and eNOS, as opposed to ASCs cultured with FGF or VEGF alone [18,78]. These results suggest a co-stimulatory effect of FGF and VEGF necessary to elicit robust ASCs differentiation into ECs.

Further information has been gathered in studies using bFGF, a 18kDa protein expressed in various cell types. bFGF has been shown to induce angiogenesis, wound healing and vascular remodeling [79,80]. The inhibition of FGF signaling in ASCs significantly reduced mRNA and protein expression of EC markers and the inhibition capacity to subsequently form capillary-like structures on Matrigel [66,78]. All these results suggest FGF as a critical inducing factor in ASCs differentiation into ECs. 

Several studies have shown the angiogenic potential of ASCs differentiated into EC-lineages as EC substitutes in vascular remodeling [81]. In these studies, ASCs cultured in specific medium for differentiation into an endothelial phenotype are subcutaneously injected into mouse models of hind limb ischemia. Subsequently, the histologic architecture, microvascular formation, capillary density, perfusion [17,82] and vascular gene expression [18] have been evaluated. The main results of these in vivo experiments showed that ASCs differentiate into the endothelial lineage and participate in blood vessel formation. It has been demonstrated by a marked increase in the blood flow as well as in the capillary density in the ischemic hind limb of nude mice [17,18,82], and interestingly ASCs can be incorporated into the new vasculature [18]. Moreover, neovascularization was confirmed by increased expression levels of angiogenic genes [81]. 

## 8. Signaling Pathways Involved in ASCs Differentiation into an Endothelial-like Phenotype

To determine the signaling pathways that regulate ASCs differentiation into ECs by EGM, the involvement of different cellular pathways has been explored. Cao et al. reported the importance of the PI_3_K pathway, one of the major pathways for EC proliferation and survival [18]. This signaling pathway is highly conserved and stimulates the phosphorylation of Akt. The activation of this protein plays a central role in numerous cellular functions including cell metabolism, growth, proliferation and survival, protein synthesis, transcription, and apoptosis. Also, it plays a central role in angiogenesis [18,83]. The results revealed that PI_3_K inhibition completely blocked ASCs-endothelial differentiation. It is important to note that this finding suggests that the endothelial phenotype acquisition is dependent on the PI_3_K signaling [18]. EGM, that is supplemented with several growth factors, such as VEGF, epidermal growth factor, insulin-like growth factor 1 and/or bFGF, stimulates the activation of the PI_3_K signaling pathway. Co-incubation of ASCs with an Akt pathway inhibitor, results in a complete abrogation of ASCs differentiation toward ECs as well as cell sprouting [16]. These findings are in accordance with the results of Cao et al. They identified VEGF/PI_3_K/Akt as a key signaling pathway for ASCs differentiation into ECs [18]. In addition, it has been demonstrated that the paracrine effects of FGF are also mediated by the PI_3_K/Akt pathway, which inhibits the activity of target molecules by phosphorylation [84].

Interestingly, Almalki et al. reported that silencing matrix metalloproteinase (MMP)-2 and MMP-14 contribute to promote an ASC-endothelial like phenotype and function after treatment with endothelial basal medium containing VEGF [77,85]. VEGF induces ASCs differentiation into ECs via the activation of VEGFR2; hence, the inhibitory effect of the MMPs could be due to the cleavage of this receptor and the inhibition of the endothelial differentiation pathway. In addition, their findings suggests that VEGF activates VEGFR2, thus activating the MAPK/ERK signaling pathway to promote and induce the transcription of EC markers during ASCs differentiation into ECs [85].

**Figure 3:**
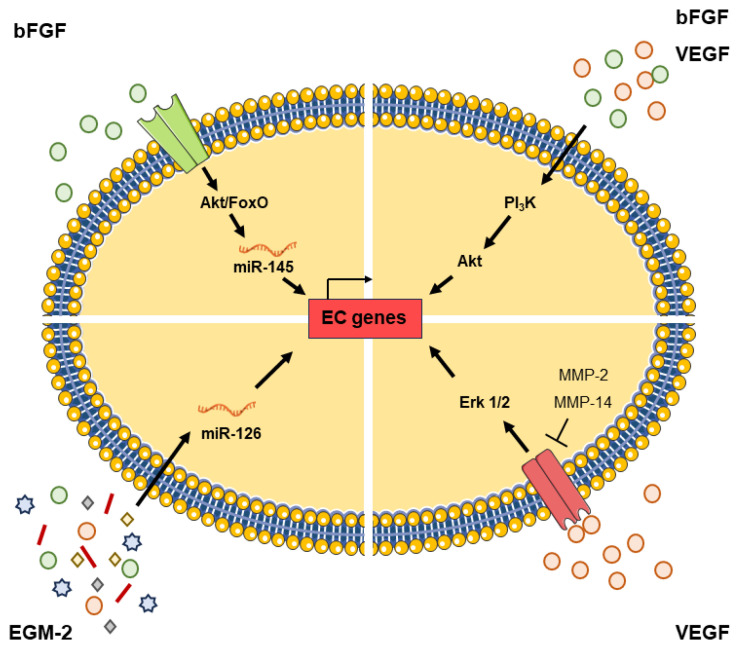
Transcriptional regulation in ASCs differentiation into ECs. ASCs endothelial differentiation into EC-like cells by the acquisition of EC phenotype is regulated by several signaling pathways, transcription factors and microRNAs. Growth factors, present in endothelial cell growth medium or secreted by ECs, interact with their receptors in ASCs. Thus, it activates PI_3_K and MAPK signaling pathways, and regulates the transcription of EC-specific cell markers, such as CD31, von Willebrand Factor, CD144, PECAM-1, CD34, and eNOS. Moreover, these factors can also regulate the expression of microRNAs (miRNA-145 and miRNA-126), which interact with transcription factors that in turn induce the up-regulation of EC markers.

On the other hand, miRNAs are short non-coding RNA molecules involved in the regulation of several cellular processes. MiRNA-126 has been identified as an endothelial-specific and highly expressed miRNA [86,87]. It is involved in the regulation of endothelial migration, cytoskeleton reorganization, capillary network stability, cell survival and apoptosis [87]. Furthermore, studies on miRNA-126 demonstrated that this miRNA is necessary for the maintenance of vascular integrity [86,88]. Xie et al. observed that during ASCs differentiation into ECs by EGM, miRNA-126 expression gradually increases as well as the expression of vascular endothelial cell markers [86]. Interestingly, Arderiu et al. focused their research in the interaction between ECs and ASCs through their secretome. They found that microvascular ECs produce and secrete bFGF, which regulates ASCs differentiation into ECs. It down-regulates miRNA-145 expression in ASCs [89] and promotes angiogenic properties and vascular network formation through targeting V-ets avian erythroblastosis virus E26 oncogene homolog 1 (ETS1), a transcription factor necessary for the maintenance of endothelial integrity and capillary formation. The intracellular signaling pathway is involved [84] (Figure 3).

## 9. Other Cells with Angiogenic Potential

Monocytes are a heterogeneous population of mononuclear cells responsible for the control and clearance of infections. In addition, it has been shown that monocytes also play an intricate role in different biological functions being one of them angiogenesis [90,91]. The initiation of neovascularization in ischemic areas is related to the infiltration and activation of inflammatory cells within hypoxic areas [28,92,93]. Activated ECs release molecules that recruit monocytes from circulation to the ischemic tissue and subsequently promote postnatal neovascularization [91]. Recruited monocytes stimulate EC functions by releasing proangiogenic mediators, but at the same time, the interaction between monocytes and ECs results in cross-modulation of angiogenesis. The proangiogenic environment induces monocytes to transdifferentiate into endothelial cell-like cells and acquire endothelial features [90,91,94].

We have contributed to clarify this interaction of endothelial cells and monocytes to promote angiogenesis [90,91,94,95]. We demonstrated that monocytes have a paracrine cross-talk with microvascular ECs with release of extracellular microvesicles (EVs) [94]. In addition, this cross-talk induces ECs to release tissue factor-rich MVs, which induces monocytes transdifferentiation into EC-like cells ready to form newly release of EVs. Therefore, there is a positive feedback between monocytes and microvascular ECs that induces angiogenesis in ischemic zones, contributing to ischemic tissue repair [91,95]. Activated ECs also release EVs containing high levels of miRNA-126, which are transferred to monocytes and while it does not promote EC-like cell differentiation it has effects on several components of angiogenic pathways [90].

## 10. Conclusions and Future Perspectives

The field of therapeutic angiogenesis was met with great excitement in the late 1990s and appeared as a radical new paradigm for treating ischemic diseases. The enthusiasm generated by those early studies was dampened by disappointing results. In recent years, stem cell transplantation has been recognized as a new technique with therapeutic angiogenic effects on ischemic diseases. However, the presence of cardiovascular risk factors and metabolic disorders in patients with PAD seems to negatively influence the effects of adult stem cells, discouraging their autologous use in the clinic. The use of allogenic stem cells from healthy donors may overcome these limitations. Different studies have proven the capacity of ASCs and their secretome as an attractive vehicle for cell-based therapeutics. The modulation of different molecular mechanism increases the therapeutic efficiency of ASCs and suggests a potential strategy to elevate their angiogenic effect for the treatment of ischemic diseases. Therefore, direct cell differentiation is a promising direction in the cell-based therapy and regenerative medicine fields, especially in ischemic tissue regeneration by therapeutic angiogenesis. Taking this into consideration, further studies are required to ascertain and differentially characterize the roles and beneficial effects of the different autocrine and paracrine regulators of angiogenesis and neovessel formation in ASC differentiation into ECs.

## Data Availability

Not applicable.
